# Preanalytical Errors in a Hematology Laboratory: An Experience from a Tertiary Care Center

**DOI:** 10.3390/diagnostics13040591

**Published:** 2023-02-06

**Authors:** Mohammad Shahid Iqbal, Aisha Tabassum, Ahmad Fawzi Arbaeen, Ahmed H. Qasem, Adel G. Elshemi, Hibah Almasmoum

**Affiliations:** 1 Department of Laboratory Medicine, College of Applied Medical Sciences, Umm al Qura University, Makkah 21955, Saudi Arabia; 2 Department of Laboratory Medicine, Faculty of Applied Medical Sciences, Umm al Qura University, Makkah 21955, Saudi Arabia

**Keywords:** hematology, laboratory, preanalytical error, quality assurance

## Abstract

Background: Laboratory errors arise at any stage of testing. Detecting these inaccuracies before results are revealed might delay diagnosis and treatment, causing patient distress. Here, we studied the preanalytical errors in a hematology laboratory. Methods: This one-year retrospective analysis was conducted at the laboratory of a tertiary care hospital and included information on blood samples that were taken for hematology tests from both outpatients and inpatients. Laboratory records included sample collection and rejection information. The type and frequency of preanalytical errors were expressed as a proportion of total errors and sample number. Microsoft Excel was utilized to enter data. The results were presented in the form of frequency tables. Results: This research included 67,892 hematology samples. For preanalytical errors, 886 samples (1.3%) were discarded. The most common preanalytical error was insufficient sample (54.17%), and the least common was an empty/damaged tube (0.4%). Erroneous samples in the emergency department were mostly insufficient and clotted, whereas pediatric sample errors were caused by insufficient and diluted samples. Conclusion: Inadequate samples and clotted samples account for the vast majority of preanalytical factors. Insufficiency and dilutional errors were most frequent from pediatric patients. Adherence to best laboratory practices can drastically cut down on preanalytical errors.

## 1. Introduction

Modern day diagnostics heavily depends on accurate laboratory test results, and hence it is necessary to ensure the reliability and accuracy of lab results [1]. A medical lab plays a crucial role in providing timely and accurate results of laboratory investigations essential for patient management [2]. One of the busiest areas of the clinical laboratory is the hematology laboratory. Hematology tests are frequently available even in tiny, limited-service laboratories [3]. Quality assurance in a hematology laboratory is necessary to ensure for laboratory users accurate test results with a high degree of precision [4]. A standardized, reliable test result is the purpose of quality assurance in the laboratory [5]. To achieve the goal of providing safe health care to the patient, quality in medical diagnostics is of utmost importance [6]. The total testing process (TTP) in a laboratory encompasses every step, from test requisition to the receipt of results [7]. In almost 70% of cases, clinical decision making depends on laboratory results, and hence accuracy and reliability of results is crucial [8]. Clinical decisions such as admission, prescription, and discharge are based on the laboratory test results, and more importance should be given to the quality of test results as they play a significant role [9]. Clinical laboratory errors can be reflected in increased healthcare costs and decreased patient satisfaction [10].

Laboratory errors are mistakes made during the entire testing process. Such errors might be due to miscommunication between laboratory personnel as well as actions performed by others in the process or might be due to a poorly designed process [11]. The laboratory errors have a significant impact and may cause a delay in diagnosis or treatment if they are detected before the issue of results, and therefore lead to patient inconvenience or anxiety, and in some cases the opportunity can be missed for diagnosis or screening if the specimen cannot be retaken. Furthermore, the errors which go undetected before the release of results will present unwanted errors and may result in the wrong diagnosis or missed diagnosis, unwanted retesting or treatment, and might put the safety of patient at risk [12].

Laboratory sample processing errors can be divided into three categories: preanalytical, analytical, and postanalytical errors [5]. The term preanalytical phase was coined by Statland and Winkel in 1977 [13]. Automation has significantly reduced the errors occurring in the analytical and postanalytical phases, whereas the preanalytical stage still has a long way to go as it is mostly dependent on manual labor [14]. Although there is plenty of automation in the laboratory processes, there are numerous variables that can affect laboratory results, which are mainly due to human intervention and hence are preventable [13,15,16,17].

For the correct reporting of results, all the three phases of the total test process need to be free of errors. During laboratory processes, a significant proportion of preanalytical errors have shown to increase patient safety risks [18]. Preanalytical activity begins from clinical request for a laboratory test to the sample preparation for analysis. A study in a 650-bed hospital in the US reported preanalytical error specimen costs between 0.23% and 1.2% of the total hospital expenditure cost, which is approximately USD 1,199,122 [2]. It has been reported that in the total testing process, only 7–13% of errors arise in the analytical phase, whereas the majority of errors occur in the preanalytical phase (46–68%) and remain in the postanalytical phase (18–47%) [7,10,17]. Although guidelines and standard protocols related to blood sampling are available, there is a low compliance for them [6]. A study from Italy reported that 62% of the laboratory errors were seen in the preanalytical phase of the TTP before the specimen reached the laboratory [12]. The safe and correct collection of blood samples is essential in a hematology laboratory, as incorrect and unsafe procedures can cause errors during analysis.

Preanalytical errors occur before the analytical phase of the TTP and can occur before or after the laboratory receives the material. They have been proven to account for a considerable fraction of laboratory errors. They raise the likelihood of erroneous or inappropriate treatment intervention, unnecessary follow-up, and diagnosis delays, as well as reduce the clinical and economic effectiveness of laboratory services [18].

Examples of errors that arise in the preanalytical phase include errors in test ordering, patient identification, patient preparation, collection of samples, quality of collected sample (diluted, clotted, and hemolyzed sample), inappropriate containers and anticoagulants, and sample transportation and storage. Sorting of sample, its centrifugation, labeling, and separation also come in the preanalytical phase [12,14]. The objective of this study was to identify the preanalytical errors and their frequency in the hematology lab.

## 2. Materials and Methods

This is a retrospective study conducted in the laboratory of a tertiary care hospital. The duration of the study was one year. The hospital laboratory receives blood samples from outpatients and inpatients. In the hospital laboratory, the blood sample collection is centralized, and samples from various sections such as hematology, microbiology, and biochemistry were collected in the main collection area by trained laboratory technicians, whereas the ward nurses collected the samples from the admitted patients. Color-coded blood collection tubes were used for sample collection. For adults, 3 mL volume capacity tubes were used, and for pediatric sample collection, 2 mL capacity non vacuum tubes were used. No microtubes were used for sample collection. For coagulation tests, blue-topped tubes containing 3.2% sodium citrate of 3 mL and 2 mL capacity were used for adults and pediatric patients, respectively. The hematology samples collected comprise all samples for routine hematology tests, such as complete blood counts, and routine hemostasis tests, such as prothrombin time and activated partial thromboplastin time. We collected all the relevant data from the laboratory records. Laboratory records included sample collection and rejection information. During the study period of one year, a total of 67,892 blood samples were collected for hematology tests. Out of these, 53,249 samples were from the outpatients and 14,643 samples were from the admitted inpatients. The preanalytical variables identified in this study that can affect the test results were sample in inappropriate collection tube, clotted blood sample, insufficient sample, hemolyzed sample, diluted sample, excess sample, empty vacutainer/damaged tube, wrong label, and delay in transfer to laboratory. For the coagulation tests, only those samples with the exact volume as per the mark on the tube were accepted, and the others were rejected and were included in the preanalytical error group. In the laboratory study groups, the type of the errors and their frequency were displayed as a percentage of both the overall number of errors and the total number of samples. Statistical methods: data were entered in Microsoft Excel 2020. Analysis was conducted and is presented as frequency tables.

## 3. Results

This is a retrospective study conducted for the data collected over a period of one year from a tertiary care hospital laboratory. A total of 67,892 samples received for hematological tests were included in this study. There were 886 (1.3%) samples that were rejected for various preanalytical errors. Insufficient sample was the most common preanalytical variable (54.17%) in this study, and empty tube/damaged tube was the least reported error (0.4%). A significant number of samples were rejected due to clotting (20.09%) (Table 1).

**Figure 1:**
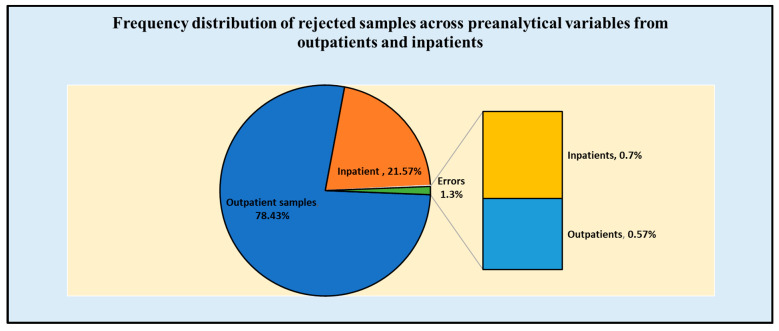
Frequency distribution of rejected samples across preanalytical variables from outpatients and inpatients.

Out of the 67,892 samples, 53,249 (78.43%) were from outpatients attending the clinics, and the remaining 14,643 (21.57%) were from the inpatients admitted in various departments of the hospital. Amongst all the erroneous samples, most preanalytical errors were from the inpatient department (0.7%), whereas the outpatient department erroneous samples comprised 0.57% of the total (Figure 1). Among the total rejected samples from the inpatient department (IPD), pediatric samples comprised the majority of erroneous samples (36.2%), followed by samples from the emergency department (26.76%) (Table 2).

Among the erroneous samples received from the emergency department, the majority were insufficient samples followed by clotted samples, whereas most insufficient samples as well as diluted samples were from the pediatrics department. Although there were no samples with delay in reaching the lab from the emergency department, errors of wrong labeling or missed labeling of samples were predominantly seen among them (Table 3).

## 4. Discussion

A laboratory error is any defect that occurs during the whole testing process, from ordering tests to reporting results, and affects the quality of laboratory services in any manner [19]. Up to seventy-five percent of laboratory errors can be attributed to errors in any of the components of the pre-testing phase [19,20]. By categorizing laboratory errors according to their severity, it is possible to determine which errors require immediate attention for quality improvement and to implement corrective/preventative measures to reduce them [21]. Laboratory errors are directly linked to cost constraints and result in poor patient satisfaction [6]. In this retrospective study of one year, we studied the frequency and types of preanalytical variables that resulted in sample rejection. Overall, there were 886 (1.3%) preanalytical variables reported from different departments in a tertiary care center. Similar proportions of errors have been reported by other studies (Table 4) [4,9,15,20].

We used collection tubes of 3 mL and 2 mL volume capacity for adults and pediatric patients, respectively. In our investigation, the majority of blood samples were rejected owing to insufficiency, wherein the proportion of blood sample to anticoagulant is incorrect. Strict criteria of rejection were followed for the sodium citrate collection tubes for coagulation studies, wherein any tube with a sample not exactly at the level of the mark were rejected. Others have also found a significant percentage of insufficient samples in their studies [17,20,22,23]. It is possible that undertrained phlebotomists and poor proficiency quality are to blame. The EDTA tubes have a set fill volume that ensures the best anticoagulant concentration, and both under- and overfilling can result in incorrect complete blood cell count readings. Even if the sample is not clotted, overfilling the sample can result in insufficient mixing prior to testing, which can result in pseudo polycythemia, pseudo thrombocytopenia, and pseudo leukopenia. Low hematocrit, low MCV, and a high MCHC are further alterations associated with insufficient volume when processed on an automated analyzer [12,24]. It has also been reported that coagulation tests such as prothrombin time (PT) are falsely prolonged in such samples [22].

In our study, the second most common preanalytical error was clotted samples comprising 20.09% of all errors. Other studies have also reported a high proportion of specimens rejected because of clotting [4,9,25]. For obvious reasons, such samples cannot be processed. Even with anticoagulated tubes, if the tube is not gently inverted four to five times to ensure uniform mixing of the anticoagulant with the blood sample, samples may clot. Most of the clotted samples in our investigation came from an inpatient department that dealt with emergency patients. This could be owing to the high workload of staff, as critical cases continue to be admitted, and employees are more prone to exhaustion, which could influence the quality and proficiency of operations such as phlebotomy. Our findings contradict those of Gaur et al. [20], who found that clotted samples were the most common among outpatient samples. This variation could be explained by a difference in our sample sizes. The most common causes of blood clotting are an insufficient anticoagulant to blood ratio or a delay in transferring blood from a syringe to a vial. Clotting causes cell damage and the consumption of coagulation factors, rendering the sample unusable for assays requiring plasma or whole blood [14]. Hemolyzed samples in our study comprised about 4.6% of the total rejected samples. A similar proportion of hemolysis has been reported in other studies [22]. Poor sampling practices such as using alcohol to clean the venipuncture site and failing to allow it to dry properly (at least 30 s), using syringes to take blood, vigorously mixing samples, and forcing blood into a tube with the syringe plunger are major causes of hemolysis [14]. It has also been proposed that coagulation reactions are expedited in hemolyzed materials [22]. Sample collection using intravenous (IV) catheters is a primary source of possible hemolysis during specimen collection. Hemolysis in serum samples taken using IV catheters is reported to be 29%, compared to 1% when a sample is acquired via straight needle venipuncture [26]. According to another study, extensive hemolysis is likely to give incorrect CBC results [27]. Delay in sample transfer was noted in 2.2% of the rejected samples. Another study also reported a similar proportion of error regarding sample transport time [17]. Ideally, all CBC specimens should be analyzed within 6 **h** of collection, particularly if blood cell morphology is necessary [28]. Due to delayed reporting of time-critical data, sample deterioration, especially for cell morphology, increased MCV, MPV, and hemolysis, there is a risk of harm to patients [12].

We also observed that in our study, sample insufficiency was more frequent among the inpatient samples when compared to outpatient samples. This is similar to other studies [9,20,29]. Delay in sample transfer to lab as well as error in the labeling of samples was more pronounced in inpatient samples (Table 5). This could be owing to the heterogenous workforce, which includes inexperienced interns and nursing staff stationed in wards, and it also indicates the need for enhanced training and education for the blood collecting staff. The information concerning the preanalytical phase might be updated by offering frequent training to all workers, and therefore it may be able to greatly reduce preanalytical errors in health practice and nursing science [30].

The most insufficient and diluted samples were collected from the pediatrics division, which was an additional finding of relevance. This may be owing to the difficulty in obtaining venous blood samples from newborns and young children, as well as the fact that capillary blood sample collection increases preanalytical mistakes [22]. Preanalytical mistakes may also be exacerbated by the collecting of materials in many tubes for different investigations. In addition, sample collection by intravenous catheters may result in diluted samples. The significant frequency of preanalytical errors in pediatric hospital care demonstrates that the blood sampling method is complex and error prone [31]. The proper labeling of vials prior to sampling is of the utmost importance because it is impossible to trace the sample source after sampling has been completed, and a mistake at this stage could lead to falsification or report swapping. In this investigation, 29 (3.2%) of the total rejected samples were incorrectly labeled. Transporting samples correctly from the collection site to the laboratory is of the utmost significance. In our analysis, 0.4% of samples were compromised or leaked. Improper transport could result in sample volume loss, sample mixing, loss of sterility, and public exposure to infectious materials [14].

Most errors, when found, result in sample rejection at the laboratory prior to processing, necessitating resampling and increasing the hospital staff’s burden, wasting hospital resources, aggravating the patient’s discomfort, and delaying the reports unnecessarily [32,33,34].

On reflection of the probable reasons for these preanalytical errors, it can be claimed that ignorance and indifference regarding optimal phlebotomy procedures and prior patient preparation on the part of sample collection personnel are at the heart of the issue. Staff training and standardization of phlebotomy procedures have been demonstrated to enhance specimen quality.

## 5. Conclusions

Preanalytical errors are still a big problem in any lab, since most of the steps that lead to these mistakes are out of the lab’s direct control. They cannot be stopped, but they can be avoided. Our study showed that most preanalytical variables are caused by samples that are too limited or have clotted, whereas diluted samples and insufficient volume were predominant errors among the pediatric group of patients. There is an indispensable need to standardize laboratories in terms of occurrence of errors in all phases of the total testing process. The vacutainer system with evacuation tubes needs to be used most effectively, samples need to be bar-coded, and staff who collect samples need to be trained regularly. Compliance with good laboratory practices can cut the number of preanalytical errors by a significant number. Knowledge of the intervening factors that can influence laboratory results, effective communication and coordination between the laboratory and the wards, as well as formal training and continuing medical education programs for laboratory and paramedical staffs can help prevent this.

## Figures and Tables

**Table 1:** Frequency distribution of rejected samples across preanalytical variables.

	Preanalytical Variables	Number of Samples N (%)
1	Inappropriate tube	108 (12.19 %)
2	Clotted sample	178 (20.09%)
3	Insufficient sample	480 (54.18%)
4	Hemolyzed sample	41 (4.63%)
5	Diluted sample	16 (1.80%)
6	Excessive sample	10 (1.13%)
7	Labeling error	29 (3.27%)
8	Delay in sample transfer to lab	20 (2.26%)
9	Empty tube/damaged tube	4 (0.45%)
	Total	886 (100%)

**Table 2:** Frequency distribution of rejected samples across preanalytical variables from outpatients and inpatients.

	Preanalytical Variables	Samples from Outpatients 53,249 (78.43%)	Samples from Inpatients 14,643 (21.57%)	Total Number of Hematology Samples 67,892 (100%)
1	Inappropriate tube	47 (12.08%)	61 (12.27%)	108 (12.18%)
2	Clotted sample	81 (20.83%)	97 (19.51%)	178 (20.09%)
3	Insufficient sample	213 (54.75%)	267 (53.72%)	480 (54.17%)
4	Hemolyzed sample	24 (6.17%)	17 (3.43%)	41 (4.60%)
5	Diluted sample	3 (0.77%)	13 (2.62%)	16 (1.80%)
6	Excessive sample	7 (1.8%)	3 (0.60%)	10 (1.10%)
7	Labeling error	11 (2.83%)	18 (3.63%)	29 (3.20%)
8	Delay in sample transfer to lab	3 (0.77%)	17 (3.42%)	20 (2.20%)
9	Empty tube/damaged tube	0 (0.00%)	4 (0.80%)	4 (0.40%)
	Total	389 (0.57%)	497 (0.73%)	886 (1.30%)

**Table 3:** Frequency distribution of preanalytical variables among the various inpatient departments.

		Inpatient Departments				Total
	Preanalytical Variables	Medicine	Surgery	Pediatrics	Emergency	
1	Inappropriate tube	15	15	2	29	61
2	Clotted sample	25	11	21	40	97
3	Insufficient sample	38	45	136	48	267
4	Hemolyzed sample	4	3	1	9	17
5	Diluted sample	1	2	10	0	13
6	Excessive sample	2	1	0	0	3
7	Labeling error	4	4	3	7	18
8	Delay in sample transfer to lab	5	6	6	0	17
9	Empty tube/damaged tube	1	2	1	0	4
		95 (19.11%)	89 (17.90%)	180 (36.22%)	133 (26.77%)	497 (100%)

**Table 4:** Comparison of preanalytical errors in hematology laboratory.

Study (Year)	Total Number of Samples	Total Errors/Error Frequency (%)	Most Common Error
Upreti et al. (2013) [15]	135,808	1339 (1%)	Wrong label, insufficient sample
Narang et al. (2014) [4]	471,006	1802 (0.38%)	Clotted, insufficient sample
Arul et al. (2018) [9]	118,732	513 (0.43%)	Insufficient sample, clotted sample
Gaur et al. (2020) [20]	189,104	4052 (2.14%)	Insufficient sample, clotted sample,
Present study	67,892	886 (1.3%)	Insufficient sample, clotted sample

**Table 5:** Comparison of error frequency and types with other studies between OPD and IPD samples.

	Arul et al. [9]		Gaur et al. [20]		Present Study	
	Number of Samples (%)		Number of Samples (%)		Number of Samples (%)	
	Outpatients	Inpatients	Outpatients	Inpatients	Outpatients	Inpatients
Unlabeled/Misidentification	9 (0.01)	14 (0.02)	27 (0.05)	110 (0.08)	11 (0.01)	18 (0.02)
Incorrect vials	28 (0.04)	32 (0.06)	5 (0.01)	0 (0.01)	47 (0.06)	61 (0.08)
Inadequate samples	104 (0.17)	131 (0.23)	422 (0.75)	1695 (1.27)	213 (0.30)	267 (0.39)
Clotted sample	67 (0.11)	72 (0.13)	815 (1.46)	857 (0.64)	79 (0.10)	97 (0.14)
Diluted sample	0	23 (0.04)	4 (0.01)	9 (0.01)	3 (0.0)	13 (0.01)
Hemolyzed sample	15 (0.02)	18 (0.03)	20 (0.04)	54 (0.04)	24 (0.03)	17 (0.02)
Excessive sample	-	-	2 (0.00)	2 (0)	7 (0.0)	3 (0.0)
Total error (%)	0.43%		2.33%		1.30%	

## Data Availability

Sample collection related data are available.
